# An interdisciplinary program for emerging leaders in patient safety

**DOI:** 10.1111/tct.13507

**Published:** 2022-05-31

**Authors:** Kim Oates, Annette Burgess, Tyler Clark

**Affiliations:** ^1^ Faculty of Medicine and Health, Sydney Medical School, Discipline of Child and Adolescent Health The University of Sydney Sydney New South Wales Australia; ^2^ Faculty of Medicine and Health, Sydney Medical School, Education Office The University of Sydney Sydney New South Wales Australia

## Abstract

**Background:**

Having previously shown that an interprofessional immersive course, AELPS (Academy for Emerging Leaders in Patient Safety) can change the way young clinicians think about patient safety, we surveyed them between 1 and 5 years later to determine its longer‐term influence on careers, relationships with colleagues and with patients.

**Methods:**

All alumni from 2016 to 2019 (*n* = 116) were invited to complete a survey on the usefulness of their AELPS experience in: obtaining their current position; doing patient safety projects; understanding and working with patients; improving communication skills; breaking down hierarchies; networking; mentoring and using new skills in the workplace. Data were analysed using descriptive statistics and thematic analysis.

**Results:**

Response rate was 56%. Over 85% reported ongoing improvement in medication safety knowledge, communication with patients, use of graded assertiveness, communicating more effectively with colleagues, seeking views of their patients about treatment options and seeing things from the patient perspective. Sixty seven per cent agreed that AELPS helped them in their career choice and 57% agreed it had helped them obtain their current position. Skills transferred to the workplace included ability to make improvements, establish education initiatives and model patient‐centred care. Stumbling blocks included a hierarchal culture and lack of accountability for patient safety practices in some hospitals.

**Discussion:**

An intensive, interdisciplinary program on patient safety can provide future health leaders with ongoing tools to improve communication, understand the patient view and speak up on behalf of the patient, all factors that contribute to improving safety of patients.

## 1 INTRODUCTION

*“Leadership has never been more important in healthcare. It is a vital factor in highly ranked healthcare organisations where capable, high‐quality leaders, who embody a collective leadership style, are essential to support high‐quality patient care*”.^1^ This is particularly so in patient safety, an area where many of the current medical leaders are senior clinicians who excelled in their own specialty before making a transition to patient safety prior to retirement. This means we will soon need a new generation of patient safety experts who have the qualities of the new style of clinical leader.^2^

Clinical leadership can be described as the ability of healthcare staff to carry out roles that use their expertise and skills to further the core values of their profession and to keep the needs of the patient as the central focus.^3^ As that definition implies, leadership is a crucial factor in making teams work in a way that builds a culture of patient safety in a way that will transform services.^4^ Leadership is not confined to the medical profession. Leadership skills should be undertaken by the person most appropriate to the situation, regardless of profession of position in the hierarchy.^5^

In 2019 we published the description and outcomes of the Academy for Emerging Leaders in Patient Safety (AELPS) held in Sydney, Australia between 2016 and 2018.^6^ Each AELPS comprised a 4‐day intensive, interactive workshop for up to 30 scholars made up of junior doctors, nurses, pharmacists and medical students, all selected for their potential as future leaders through a competitive process. The 10 faculty included clinician patient safety experts and patient advocates. The emphasis was on informality, lack of hierarchy, working alongside and learning from other health professionals in a program involving discussions, interactive presentations, storytelling, graded assertiveness exercises and communication skills. Our previous study demonstrated a positive shift in participant attitudes towards teamwork, patient centredness and self‐care with appreciation of the interprofessional nature of the Academy, opportunities provided for networking and a structure that allowed interaction with patient advocates and faculty members.

This current study sought to determine the longer‐term influence of attending the Academy in terms of career pathways, relationships with colleagues and relationships with patients.

We sought to determine the influence of the Academy on career pathways and relationships with colleagues and patients.

## 2 METHODS

### 2.1 Data collection and analysis

In 2020, all alumni from 2016 to 2019 (n = 116) were invited to voluntarily complete an anonymous online survey. As researchers, we recognise that faculty development programs alone do not create change. Rather, participants are introduced to the resources and opportunities for change. In exploring the long‐term impact of the Academy, we therefore adopted a realist approach to consider what works for whom within the contexts of their workplace, and within their capacity.^7^

The survey included 18 closed items, using a 5‐point scale, ranging from ‘strongly disagree’ to ‘strongly agree’. The survey items are shown in Appendix A. They focussed on the usefulness of their AELPS experience currently in: obtaining their current position; understanding patient safety and patient safety projects; understanding and working with patients; improving communication skills; breaking down hierarchy within and between professions; networking; mentoring and using new skills in the workplace. A supplemental free text option was available for two of these items. Alumni were also asked to respond to four open‐ended questions about how the Academy may have helped them and any stumbling blocks they may have encountered.

Quantitative data were analysed using descriptive statistics. Qualitative data were analysed inductively using thematic analysis.^8^

## 3 RESULTS

Between 2016 and 2019, 116 health professionals completed the AELPS, including 41 (35%) junior doctors, 34 (29%) early career nurses, 32 (28%) senior medical students and nine (1%) pharmacists. Of the 116 participants, 70 (60%) were female. Of the participants, 65/116 (56%) responded to the survey: 23 (35%) junior doctors, 16 (24%) nurses, 17 (26%) senior medical students and eight (12%) pharmacists. Of the 65 respondents, 38 (58%) were female.

### 3.1 Responses to closed items

Responses are reported across 4 themes:

1. Impact on career progression since attending the Academy,
2. Improvement in professional knowledge and performance,
3. Improved relationships with colleagues,
4. Better understanding of the needs and views of patients.

1. Impact on career progression:

Sixty seven percent agreed or strongly agreed with the statement that the Academy helped them in their career choice or choice of current position. Fifty seven percent agreed that the Academy assisted them in obtaining that current position.

- Impact on improvement in professional knowledge and performance:

There were five statements on this theme. One hundred percent agreed that their understanding of patient safety increased, 91% were now more aware of medication safety, 87% were more likely to use graded assertiveness techniques in speaking up to seniors about patient safety issues, 74% had been given opportunities in their work to demonstrate their new skills and 59% had taken on clinical practice improvement projects.

- Improved relationships with colleagues:

Ninety seven percent agreed that they could now communicate more effectively with colleagues, with over two thirds agreeing with the other statements in this theme, the lowest response being a 67% agreement that attendance had helped them making useful networks with others.

- Better understanding of the needs and views of patients:

The most favourable results were in this theme where over 90% of respondents agreed with the 4 statements about now being able to communicate more effectively with patients and their families, being more likely to see things from the patient's viewpoint, to seek the views of patients and involve them as partners in their own care.

**FIGURE 1:**
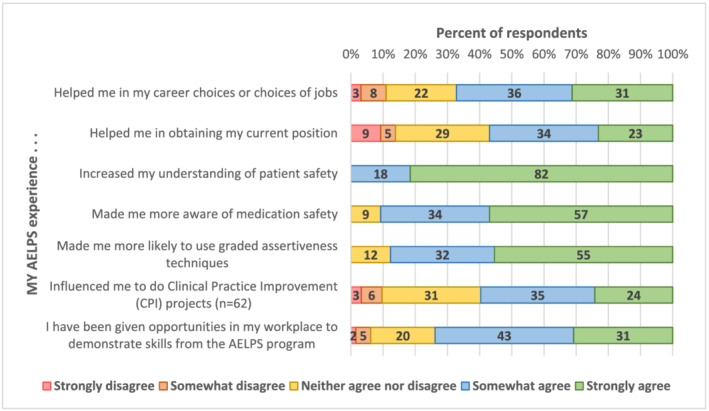
Themes I and 2: Impact of 2016–2019 AELPS participation on career (*N* = 65)

**FIGURE 2:**
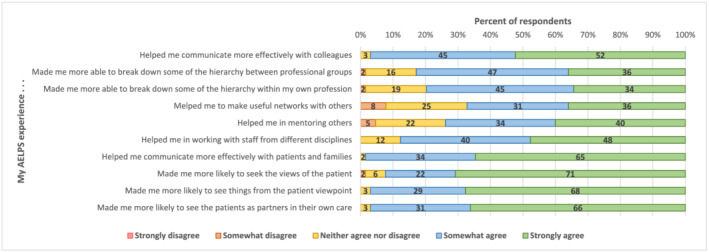
Themes 3 and 4: Impact of 2016–2019 AELPS participation on professional relationships (*N* = 65)

The responses to these 4 themes, showing the percentages in each of the 4 response categories are shown in Figures 1 and 2. The final statement not included in these four themes, “I would you recommend AELPS to others” had 100% agreement.

Over 90% were now more able to communicate more effectively with patients and their families.

To determine whether responses were consistent across the 4 years of the Academy, an analysis of variance (ANOVA) was conducted on respondent's mean item score, corrected for the number of questions answered. This found no evidence of a difference of AELPS experience depending on the year the program was attended (*F* = 0.254, *df* = 3, *p* = 0.86).

To determine whether responses differed by profession regarding their survey responses, the 3 largest groups, each of relatively equal size (medical students, nurses, and junior doctors) were compared. There was no evidence of a difference in overall corrected mean response values across these three groups (*F* = 1.704, *df* = 2, *p* = 0.19).

### 3.2 Responses to open‐ended questions

Participant responses to open‐ended questions are displayed in Tables 1, 2, 3. Table 1 shows responses to Question 1: What were the most relevant aspect of the AELPS that have helped or influenced choices you have made in your career so far? Responses indicated that the elements taught during the AELPS that had been influential included putting emphasis on the patient perspective, how understanding systems can lead to change and reduce error and the importance of interdisciplinary teamwork.

**TABLE 1:** Most relevant aspect of the AELPS that have helped or influenced choices made in careers

Theme	Examples of participant comments
The emphasis placed on the **patient perspective** and the use of storytelling during the AELPS	*Patient centred care. Whether clinical or teaching a standard of care I draw my experience from AELPS and remember the families of patients lost. Complacency kills. Its my motto*.
	*Given me a better appreciation for patient and their carers perspectives of being admitted to hospital. The course utilised the power of storytelling and this was very thought provoking, reflective and inspirational. While I had done some quality improvement prior to the AELPS this course really helped me put patient safety as the key priority and shaped the roles that I then ultimately filled*.
	*The course really helped me put patient safety as the key priority and shaped the roles I have subsequently filled*
The emphasis on preventing medical errors, **understanding how systems are used**	*The AELPS program has provided me with a heightened awareness of medical errors and the ways in which we can work to improve the quality of care we provide*.
	*Understanding the range of human factors that contribute towards patient safety. Taking time to reflect and consider alternate viewpoints ‐ particularly patients, their families and other colleagues. I went into internship feeling more prepared and knowledgeable when it came to patient safety, system issues leading to error, and how to openly and compassionately communicate with patients and their loved ones in the event of an error. The information provided on systems errors and human factors engineering was eye opening and has helped me in decision making throughout my career so far*.
The open discussion with **senior staff and patient advocates**	*The open discussion with Senior healthcare staff and patient advocates. I will never forget this. Hearing from leaders in healthcare has made me more aware of the importance of leadership in fostering a culture of patient safety. The teamwork exercise for medication safety; meeting people from the higher echelons of health; & hearing from these experts, including patient advocates*.
	*Hearing from patient advocates and families has led me to spend more time discussing diagnoses and management plans with patients and their families, answering questions, and exploring how the hospital experience is affecting them*.
The message that it is the **responsibility of all clinicians** to improve patient safety	*ALEPS has helped me to want to improve staff culture and safety in a bid to improve health outcomes for patients. ALEPS has also taught me that it is our responsibility as clinicians to be the voice of change and the advocate for our staff and patients. I have become the voice of staff wellbeing in my emergency department went on to build and setup a masters‐level fellowship program in a State health department t as a result of AELPS. It has embodied the same culture as AELPS, and been equally successful in bringing multiple clinical streams together around a common goal to improve the safety and quality of healthcare*.
Professional networks that were developed and maintained	*The professional network, including both attendees and mentors/faculty staff. The secluded and restful location added to the relationship building*.
	*The program created connections between passionate junior staff in with similarly passionate and extraordinary leaders. This was directly linked to my subsequent career journey which would not have been possible without these relationships being fostered as part of the program*.
Embedded the important role of each person within **interdisciplinary teams** to ensure patient safety	*Cemented my belief in the importance of interdisciplinary teams and my place within them*
	*The opportunity to engage with other health professionals on an even footing has made me more confident when interacting with all disciplines and levels in the workplace. We pharmacists often feel like we arent allowed to be in the middle of a patient care team and instead our job is to call out recommendations or warnings from the sidelines (while the doctors and nurses make the decisions and kick the goals). Engaging with doctors and nurses from different positions and career levels and knowing that in that space my voice at the patient safety table was just as valuable as theirs, helped me to feel the same way when I was back in a hospital environment*.
**Frameworks** to use in the workplace and promotion of a flat hierarchy to encourage others to **speak up**	*Graded assertiveness, team structures/power gradients, critical thinking skills, how to communicate effectively, how to escalate for patient safety*
	*We promote a flat hierarchy, always striving to create the psychological safety for any team member to speak up and ‘stop the line’ if necessary (and if it werent necessary, they are congratulated for speaking up regardless)*.

**TABLE 2:** Demonstration of knowledge, skills and tools from the AELPS, creation of networks in your workplace and beyond; and changes fostered relating patient safety?

Theme	Examples of participant comments
**Improve systems to review incidents and deaths**, Co‐ordinating clinical audits & engaging in quality improvement projects	*I utilise the knowledge and skills from AELPS in my role to improve the way we review clinical incidents, review deaths in hospital, and action our responses to clinical indicators*.
	*As a quality and safety representative ‐ coordinating multiple audits across the hospital network*.
	*Participation in clinical audits for quality improvement*.
Inspired **further studies**	*It has inspired me to further extend my learning and I am currently completing the Harvard Medical School Safety, Quality, Informatics and Leadership Course*
	*I am pursuing a Masters in Patient Safety and Healthcare Quality through Johns Hopkins and have tried to model patient‐centred care for my junior staff. Ive also had the opportunity to present to trainees about patient safety concepts and approaches*.
	*Enrolling staff into post‐graduate courses, Upskilling staff in palliative education so that as a general medical ward we could become specialist in this area. This was important to me as there are no palliative care beds available in my rural centre*.
A **greater understanding of patient safety theories**, enables engagement in practice	*By understanding the theory of patient safety, medication safety and graded assertiveness Ive felt more able to practice putting it into practice, honing my skills, and starting conversations about it with colleagues*.
	*I have applied some of my learnings and renewed passion with some multidisciplinary projects that I was already involved in, and in the orientation/education I provide to medical interns*.
**Patient‐centred behaviours**, eg. using the patients name when discussing the patient, which influences team behaviour	*Following AELPS I began to always use my patients name in discussions to make it clear that my team are also concerned about the patient This practice has spread to my broader team*.
	I *have felt more confident and comfortable in providing open disclosure to patients when mistakes have occurred, and in participating in risk analyses to identify contributing factors and improve patient safety*.
Increased **teaching capacity on patient safety**	*I often informally discuss aspects of quality and safety with medical students, residents and registrars and try to get them thinking about these concepts and the way they treat patients. Cognitive bias is a topic I am particularly interested in and have run sessions on that*.
	*I have the registrars critically analysing their medication prescriptions with regular audits*.
**Collaborated with other departments**/interprofessional on patient safety projects	*I have collaborated with the clinical pharmacy department on medication safety projects*.
	*I have worked closely with other Nurse Unit Managers, and other leaders in clinical practice to develop and implement strategies that will allow us to provide safer care*, i.e. *falls prevention, leader rounding, safety huddles, shift forecasts, problem solving as a team*.
Created **new networks locally & internationally** through presentations & development of fellowship programs	*The Fellowship program that I co‐ built with a small faculty of 3, following AELPS, has been a vehicle for developing local and international networks both within and outside our health system*.
	*I have presented our work at international conferences. I always open presentations on our program by linking to the AELPS as the starting point for ‘our journey’. Functional and ongoing networking has resulted from these conferences. In particular, we focus on the AELPS approach: Interdisciplinary, collaborative, non‐hierarchical and front‐line focused*.
Development of a community of practice patient safety programs	*The formation of a faculty for programs and a community of practice. This work has led to a large community of practice (approximately 800 and growing spanning management and state‐wide safety staff to front‐line clinicians). We actively challenge current ways of thinking through our networks and continue to build programs to bridge capability gaps*.
	*I obtained a secondment to the Australian Commission in Quality and Safety in Health Care to develop a national clinical care standard and relevant tools keep in touch with two fellow participants (both now Medical Consultants) and discuss leadership initiatives at our hospitals*
Workplace patient safety initiatives	I used skills in ongoing projects impacting on safety and quality. I received a chief executive award for a project on medication safety.
	*I managed to organise a series of identical three‐hour workshops for 40 trainees in General Medicine on person‐centred care, where the highlight was our consumer, who spoke beautifully about an orthopaedic intern who had made all the difference to her partner when he was an inpatient. I was really proud of that work. No one asked us to do it, none of my consultant colleagues knew it was happening and I received no credit for it. It was purely for the purpose of proselytising the lessons we learned at AELPS, hoping to ‘red pill’ those young doctors and let them see the ways in which our health system needs to change*.
Mentoring & educating staff	*In mentoring new staff and also in day to day work when incidents occur, being able to reflect and provide feedback to improve practice*. *I have been able to support staff to recognise/identify systems issues and provide them with a safe escalation process with “no blame” and this has enabled staff to develop and implement systems that are realistic and evidence based in preventing patient safety incidents*.
	*AELPS afforded me the opportunity to present to the board on my learnings and teach patient safety practices to staff during educational seminars. In the workplace I have since chased opportunity to innovate practice and work with open minded individuals to achieve these goals together*.
	*I believe setting a good example to my junior colleagues to take particular care when trying to multitask (especially when redoing medication tasks) and encouraging a supportive environment where my junior team members feel safe to speak up about concerns they may have with patient care*.
Use of **frameworks in the workplace** to improve communication skills	*At AELPS I learnt verbal and non‐verbal communication skills, how to implement them in the work environment and graded assertiveness which has aided by ability to communicate with my peers, patients, their family and network within my health district*.
	*Having this background allows me to voice my opinion in committee meetings. It is also an avenue for others to approach me with their concerns that I may not be aware of. Ultimately it has helped with open communication in the workplace*
**Increased responsibility** in relevant patient safety roles.	*I now chair our Safety & Quality Committee and commence each meeting with a patient safety video to set the focus on patient safety; we also have an active item each agenda where we create a plan for improvement so that we are actively contributing in addition to formal business*.
Increased adherence, and **role modelling of safety protocols** and reinforcement to other staff, particularly in more remote sites	*Increased usage of checklists. I verbalise my plan A, B, C and D before every case. This is particularly relevant in rural sites where the team is small and additional resources few. This has been incredibly well received and Ive had numerous comments like “no one has ever done this before. I feel much more comfortable knowing what were doing”*
	*Greater ability to utilise graded assertiveness and closed loop communication. Questioning current practices that are well‐established but problematic, and escalating concerns to relevant parties*.
Supporting staff in new processes, no blame culture	*Through my role in education, I have been able to empower staff to want to be safe and efficient clinicians and to value how our every action impacts on patients and their outcomes*.
	*Presented a Grand Round on adverse outcomes experienced by patients with limited English abilities*.
The introduction of new interprofessional patient safety initiatives	*Introduction of a hospital pharmacist clinical service in surgical and emergency medicine. Introduction of pharmacist prescribing into a rural hospital service. Teaching patient safety to medical students*.
	*Led a few projects on medication safety, incorporated human factors, led a project on collecting patient experiences in LHD*.
	*Clinical simulations with involvement of medical and nursing staff which have never been done here before*.
**Changes in approaches to day‐to‐day work, empowering others**	*Greater emphasis on patient safety in day‐to‐day work. Fostering a mindset of patient safety with students from their first day of working. Participating in medication safety project to promote the use of a second check. Organising infection control training for orderlies in the hospital as this was recognised as a risk to patient safety*.
	*Ensuring that as my health service transitioned to Electronic Medical Record, checklists were built in to the template notes used by junior medical staff during rushed ward rounds that would pick up on things such as missed chemothromboprophylaxis, diet/fasting status and regular medications*.
**Greater engagement and collaboration** with all staff in patient safety	*Encourage and participate in departmental audits and medication safety programs. I have done my best to empower the nursing staff to voice when they are uncomfortable with the status of a patient*
	*Every morning a snippet of safety bite issues are discussed. There are regular team motivational talks, we celebrate team success and encourage one another to think safety when doing their work. Staff morale has gone up and there is less power differential amongst the team members*.
	*I have placed more emphasis on drawing from the expertise of everyone in our team rather than trying to answer every question on my own*.

**TABLE 3:** Stumbling blocks encountered

Different rules and culture in different Australian states, Limited resources in regional areas, and poor patient safety cultures	*Where I work, often in remote locations, there is a VERY different culture in healthcare, with many clinicians, including senior ones and administrators having never even heard of open disclosure, and in many instances a culture of less accountability to clients and often negative/toxic interprofessional culture. These are all barriers to implementing a patient safety culture. However, my AELPS training has still been beneficial in better empowering me to do so*.
	*Yes, moving from a large city to a regional area has been challenging. Things are very different in regional areas with a lack of resources. Prior to starting as the Nurse Unit Manager on the ward in a regional hospital 18 months ago there was a very poor safety culture on the ward. We have made massive improvements in this space which has been positive*.
The view that patient safety is not the responsibility of every clinician, but is a **reaction** when errors occur	*Mostly the widespread perception of patient safety as a punitive, reactive process that is done by people external to their unit. There is not a lot of integration amongst the clinical staff of “patient safety” as a concept we all value. The term itself often elicits a shudder or a yawn but when you probe deeper the people value the same things patient safety services do: good patient care*.
**Lack of response and engagement in evidence‐based change**, the persistence old ways, and a lack of response for junior health professionals	*Absolutely. The “weve always done it this way” mentality is alive and strong and the hierarchy in health is too*
	*Doctors who dont like being challenged*
	*Age. Can be difficult at certain times to be taken seriously if you are a younger leader*
Differences between disciplines	*I have found and continue to find it difficult to be treated as an equal with medical practitioners, especially those who do not work in multidisciplinary environments of a hospital*
Lack of adherence to established patient safety systems	*Lots. There are colleagues who dont find checklists exciting and think that quality is something that nurses with bad backs do on Wednesdays in an office somewhere*.
	*The patient safety revolution is slow some days. More of an evolution*.
	*Its a challenge balancing safety with routines and entrenched practices that are purely to make clinicians life comfortable. There is always a battle somewhere*.
Entrenched hierarchies, and a lack of engagement in patient safety from senior consultants	*Entrenched hierarchies are hard to break down. This is particularly true for some specialties (*e.g. *surgery). But AELPS has shown me that there is a gold standard that should be pursued and accepting the status quo is not in our patients best interests*.
	*Hierarchy and bullying in medicine, very difficult to navigate even in patient safety issues. Older consultants often not as engaged in patient safety issues*
As a junior health professional, challenges include hierarchies and increased	*It can be difficult to implement changes at a junior doctor level for a variety of reasons, including but not limited to interesting/difficult team (doctor and interdisciplinary) dynamics, strong hierarchies where approaching more senior colleagues is near impossible, being overworked and not having time to identify issues and facilitate change, and self‐doubt due to lack of experience. I feel that with more experience I will be better placed to facilitate change and improve patient safety, particularly at a system level*.
	*The workload placed on junior staff with increasing paperwork can lead to demotivation and tired/disinterested staff, which carries a risk. I believe this is a wider system issue that will need to be gradually addressed*.
For junior health professionals, challenges include a lack of inclusion in committees, job insecurities, and short rotations	*There are still major barriers for more junior staff to effect change, including not being included in committees (*e.g. *Morbidity & Mortality or risk committees), rotating frequently through teams (with limited opportunity to shift long‐standing and entrenched cultures), and job insecurity (only 12‐month contracts with a culture that threatens to punish those who stick their head above the parapet)*.
	*I have felt disempowered many times, only able to identify concerns and escalate them to a more senior person, many times without ever getting any feedback, or having the feedback that no change would be possible. I have sought to acquaintance myself with senior clinicians who are solution‐oriented, who have influence in the workplace, so I can learn from their examples*.
	*Frequent changes in rotations during junior medical training prevents buy in and involvement in quality improvement activities. Whilst we may observe issues, we often are not in a team for long enough to feel comfortable to raise process improvement issues*.

Table 2 presents participant combined responses to Questions 2 and 3: **“**In what ways have you been able to share or demonstrate your knowledge, skills and tools from the AELPS, and create networks in your workplace and/or beyond your workplace?”, and “In what ways have you been able to foster change in your workplace/s related to patient safety?” In summary, participants reported that the AELPS has inspired future studies in the field of patient safety, significant contributions and improvements made to workplace systems, role modelling of patient‐centred care, commitment to teaching and mentoring in patient safety practices, establishment of patient safety education initiatives, increased networks.

Table 3 presents participant responses to Question 4, “Have you encouraged any stumbling blocks?” Difficulties highlighted included a hierarchal culture, lack of inclusion of junior health professionals in committees, poor adherence to patient safety systems in some regional districts, a lack of accountability for patient safety practices.

## 4 DISCUSSION

We know from anecdotal feedback that AELPS alumni enjoyed it immensely. But a warm feeling after attending a course is of little value if it does not result in change. This study, focussing on subsequent career progression, showed alumni were influenced in their career choice and current position, “My subsequent career journey would not have been possible without the relationships I fostered and the mentoring I received as a result of the program”; had a better understanding of patient safety, “The information on system errors and human factors engineering was eye opening and has helped me in decision making”; and were more confident in speaking up about safety matters on behalf of the patient and were now communicating better with colleagues, “I am now more confident in when interacting with all disciplines and all levels in my workplace” “It has taught me that it is our responsibility as clinicians to be the voice of change and the advocate for our staff and patients”. Importantly, they had a better understanding of the view of their patients and were communicating more effectively with them, “I now take time to find out what is important to our patients”.

One Alumnus said: ‘I now take time to find out what is important to my patients’.

Barriers included feeling unheard as a junior health professional “It can sometimes be difficult to be taken seriously if you are a junior leader”; with some alumni identifying a lack of adherence to evidence‐based systems “Entrenched hierarchies are hard to break down, but AELPS has shown me a gold standard to pursue and that accepting the status quo is not in our patients' best interests”; and poor understanding “There are colleagues who still think that quality is something nurses with bad backs do on Wednesdays in an office somewhere”. These results were consistent across each year of AELPS and across the professional disciplines of alumni.

These results are consistent with data about effective leadership courses for clinicians. A review of 45 studies on physician leadership found the important gaps in most programs to be integrating nonphysicians with physicians and limited use of interactive learning and feedback,^9^ areas that were a focus of AELPS. A recent review of leadership literature found nine attributes that support change in the practice of healthcare: motivating others; managing abuse of power and social influence; assuring psychological safety; enhancing communication and information sharing; generating a learning organisation; instilling a cooperative mindset; cultivating teamwork; fostering emerging leaders and encouraging boundary spanning.^10^ These nine attributes are all found in the AELPS program.^6^

The results raise the question of why AELPS was so well regarded and why it made a difference. Reasons may include: patient advocates were a key component of the program allowing their voices to be heard and their views understood, this being a new experience for most of the scholars; faculty members were passionate about improving patient safety and the patient experience; lack of any formal hierarchy with scholars, faculty and patient advocates being on an equal footing in terms of contributing and learning from each other and a program where nurses, medical students, pharmacists and junior doctors worked alongside each other, resulting in improved respect between the professions.

Two Alumni have now joined the AELPS faculty, one having moved to a senior leadership position in his home city, responsible for training Patient Safety Fellows in a state‐wide program. Although Covid restrictions combined with reduced funding put the program on hold for 2 years, it is now being replicated in another Australian state. Our alumni continue to communicate with and learn from through the networks they have been able to establish with each other and with AELPS faculty. Although we want these changes to continue, our alumni are at the start of their careers and will be exposed to many other influences. They will need good mentors to advise them as they keep learning how to mentor others. This is why the networks they have established, including ongoing contact with faculty will continue to be important.

One alumnus has moved to a senior leadership responsible for training patient Safety Fellows.

### 4.1 Limitations

With a response rate of 56% we may not have an accurate picture of the entire cohort, although based on the percentage of responders from each professional group there are similarities. The higher percentage of responding pharmacists may be due to their smaller overall number as pharmacists did not attend the first AELPS and that they are a cohesive group who have kept in touch. The follow up of 2–5 years, does not allow us to predict the long‐term impact of AELPS as alumni are still relatively junior, meaning some may not yet have had opportunities to assume leadership roles whereas others may abandon these concepts. All scholars were chosen for their potential leadership, so it could be argued that some may have moved to leadership roles anyhow. However, what is gratifying is that the leadership roles many have subsequently taken are in patient safety. It is here that they seem to have been most influenced.

## 5 CONCLUSIONS

This study has shown that a short, intensive, interdisciplinary program that includes interaction with patient advocates can provide future health leaders with tools to improve communication, understand the patient view, work respectfully with other disciplines and speak up on behalf of the patient, all factors that contribute to build capacity of the health professional workforce. The AELPS was able to influence health professionals who are seen as future leaders, towards leadership in a direction they may not have initially chosen: the area of patient safety where a new generation of leaders is needed.

AELPS was able to influence health professionals who are seen as future leaders, towards leadership in a direction they may not have initially chosen.

## CONFLICT OF INTEREST

None of the authors have competing intersts.

## ETHICS STATEMENT

The study was approved by the Human Research Ethics Committee of Sydney University: Evaluation of the roundtable for emerging leaders, approval number 2016/160.

## APPENDIX A

Survey items

Please provide response to the below items.

(Strongly disagree Disagree Neutral Agree Strongly agree)

Participation in the Academy

1. Helped me in my career choices or choices of jobs
2. Helped me in obtaining my current position
3. Increased my understanding of patient safety
4. Made me more aware of medical safety
5. Made me more likely to use graded assertiveness techniques
6. Influenced me to do Clinical Practice Improvement (CPI) projects
7. I have been given opportunities in my workplace to demonstrate skills from the AELPS program
8. Helped me communicate more effectively with colleagues
9. Made me more able to break down some of the hierarchy between professional groups
10. Made me more able to break down some of the hierarchy within my own profession
11. Helped me to make useful networks with others
12. Helped me in mentoring others
13. Helped me in working with staff from different disciplines
14. Helped me communicate more effectively with patients and families
15. Made me more likely to see things from the patient viewpoint
16. Made me more likely to see the patients as partners in their own care

Open ended questions:

What were the most relevant aspect of the AELPS that have helped or influenced choices you have made in your career so far?
In what ways have you been able to share or demonstrate your knowledge, skills and tools from the AELPS, and create networks in your workplace and/or beyond your workplace?
In what ways have you been able to foster change in your workplace/s related to patient safety?
Have you encouraged any stumbling blocks?
